# Global tropical cyclone precipitation scaling with sea surface temperature

**DOI:** 10.1038/s41612-023-00391-6

**Published:** 2023-06-05

**Authors:** Alyssa M. Stansfield, Kevin A. Reed

**Affiliations:** 1 School of Marine and Atmospheric Sciences, Stony Brook University, 100 Nicolls Rd, Stony Brook, 11794 NY USA; 2 Department of Atmospheric Science, Colorado State University, 3915 Laporte Ave., Fort Collins, 80521 CO USA

**Keywords:** Atmospheric science, Climate and Earth system modelling, Projection and prediction

## Abstract

Understanding the relationship between tropical cyclone (TC) precipitation and sea surface temperature (SST) is essential for both TC hazard forecasting and projecting how these hazards will change in the future due to climate change. This work untangles how global TC precipitation is impacted by present-day SST variability (known as apparent scaling) and by long-term changes in SST caused by climate change (known as climate scaling). A variety of datasets are used including precipitation and SST observations, realistic climate model simulations, and idealized climate model simulations. The apparent scaling rates depend on precipitation metric; examples shown here have ranges of 6.1 to 9.5% per K versus 5.9 to 9.8% per K for two different metrics. The climate scaling is estimated at about 5% per K, which is slightly less than the atmospheric moisture scaling based on thermodynamic principles of about 7% per K (i.e., the Clausius–Clapeyron scaling). The apparent scaling is greater than the climate scaling, which implies that the relationship between TC precipitation and present-day SST variability should not be used to project the long-term response of TC precipitation to climate change.

## Introduction

Flooding, especially flash flooding from heavy precipitation, is one of the deadliest weather events worldwide^1,2^. Understanding future changes in precipitation is essential for climate adaptation planning; however, changes in extreme precipitation are complicated by the variety of factors that influence it. The theoretical basis for understanding the extreme precipitation response to temperature change is the Clausius–Clapeyron (C-C) relationship, which shows that the saturation vapor pressure of water increases with temperature at about 7% per K at surface temperatures common on Earth. Because extreme precipitation typically occurs in saturated atmospheric environments, increases in extreme precipitation with temperature are expected to approximately follow this scaling as well^3,4^. However, some modeling and observational studies have found increases exceeding C-C for sub-daily time scales over certain areas^5–7^. Storm type, season, and large-scale circulations have been demonstrated to impact the extreme precipitation-temperature scaling^8,9^.

Investigating how extreme precipitation produced by specific storm types, such as tropical cyclones (TCs), responds to temperature change can aid in both forecasting precipitation before TC landfalls and understanding future projections of TC precipitation from climate models. Some studies of individual hurricanes have found precipitation increases greater than C-C using both models and observations^10–12^. The impacts of dynamical changes, such as changes in TC intensity or outer size, on extreme precipitation scaling are more uncertain than the impacts of thermodynamics (i.e., the C-C relationship)^13^. There is also uncertainty in how microphysical effects, such as precipitation efficiency, may change with climate warming^14^. Studying the present-day variability between extreme precipitation and temperature (i.e., the apparent scaling) is one way to explore these uncertainties, but it is unclear how the apparent scaling relates to the long-term relationship between climate warming and extreme precipitation change (i.e., the climate scaling)^9^.

The study of the observed links between extreme precipitation and temperature over the tropical oceans is a relatively new area of research, inhibited historically by a lack of long-term, sub-daily, high-resolution observations. Using precipitation data from the Tropical Rainfall Measurement Mission (TRMM) and two reanalysis products, Wang et al.^15^ found that the daily precipitation above the 99th percentile over most of the tropical oceans increased with temperature up to 25–30 ^∘^C and then decreased at higher temperatures. Other studies also found negative extreme precipitation changes with increasing temperature in the tropics, although they used gauge precipitation data and therefore studied land regions^16,17^. Over the ocean, the scaling is not likely to be solely limited by moisture availability as it can be over land^18^. A recent study that used precipitation data from two reanalysis datasets found positive apparent scaling rates of hourly 99th percentile precipitation over most of the tropical oceans but some negative rates along the equator in the Atlantic and Indian Oceans and in the subtropics^19^.

Using 3-hourly TRMM data and hourly surface temperature data from the European Centre for Medium-Range Weather Forecasting ReAnalysis (ERA5) dataset^20^, Traxl et al.^21^ also found large spatial and seasonal heterogeneity in the apparent scaling of extreme precipitation over the tropical oceans, with large deviations from the C-C scaling in both positive and negative directions. They relate the large negative scaling values over tropical oceans to a decline in surface temperature starting about 24 hours before an individual precipitation event. One possible explanation for this effect that they suggest is cooling by TCs, since the broad TC wind fields and large-scale cirrus cloud cover lead to surface temperature cooling that precedes the storm itself and its heaviest precipitation rates^21^. While negative apparent scaling rates appear in these datasets over tropical oceans at sub-daily time scales, they are spatially variable, and again this relationship may not directly translate to the climate scaling.

Since the observational record of precipitation over tropical oceans is relatively short, many studies turn to models to investigate the long-term climate scaling between extreme precipitation and temperature in the tropics. O’Gorman^22^ utilized Special Sensor Microwave Imager (SSM/I) precipitation observations for 1991–2008 to constrain global climate model projections of future tropical precipitation changes. This study used the interannual variability in the response of tropical precipitation to surface temperature changes in the observations to obtain an optimal estimate of the response of the 99.9th percentile of daily precipitation to climate change in the models, which was estimated at 10% per K with a 90% confidence interval of 6–14% per K averaged over tropical land and oceans. Other studies using global climate models have found very similar estimates of the mean climate scaling in the tropics^15,23^. Two studies using cloud-resolving models both found that heavy precipitation (the 90th percentile and above) in tropical environments increases generally follow the C-C scaling and that the strongest updrafts get stronger, despite using different model set-ups and analysis methodology^24,25^. Using a quasi-global aquaplanet with horizontal grid spacing of 12 km, one study found a mean 3-hourly 99th percentile precipitation rate response in the tropics of 7.3% per K^26^.

It is important to note that TCs did not form in any of these idealized model simulations and therefore any of their impacts on the scaling of extreme precipitation were not included. High-resolution simulation of TC precipitation could influence the climate scaling rates determined using these idealized simulations and relatively low-resolution global climate models. This is supported by a recent study that calculated the percentage change in mean 3-hourly TC precipitation rates from TRMM for 1998 to 2016 and then divided that value by the change in global mean sea surface temperature (SST) over that same time period. They found an increase of about 21% for a SST change of only 0.21 K, greatly exceeding the C-C rate^27^. However, another study that used a High-Resolution Precipitation Climate Data Record derived from the PERSIANN Dynamic Infrared Rain rate model (PDIR) found a 7.6% per K increase in mean 3-hourly TC precipitation rates over the same time period^28^. Most other studies that estimate the climate scaling of TC precipitation calculate the change in mean TC precipitation between climate model simulations of the current and future climate and then divide by basin-wide SST change^29–32^. Knutson et al.^33^ summarized 21 studies that looked at projected future changes in TC precipitation and found that the % per K estimates varied greatly, depending on model parameters, climate projection scenario, ocean basin, and precipitation metric. Overall, they estimated a best-guess global change of 14% per 2 K increase in SST, close to the C-C rate.

The variations in the estimates from different studies emphasize the importance of understanding the roles of dynamics in the extreme precipitation-temperature relationship^34^. Looking at the HiFLOR climate model under the Representative Concentration Pathway 4.5 (RCP4.5) scenario, Liu et al.^32^ found an increase in the mean precipitation rates within 100 km of the TCs’ centers of between 13% per K and 17% per K, dependent on ocean basin. They determined that when the TCs were split into different intensity categories, the super C-C rates diminished to near C-C rates in each intensity category, suggesting that the super C-C rates are driven by the 2.0–5.4% increase in TC intensity per K of SST warming. Stansfield and Reed^13^ estimated that increases in TC intensity contributed about 20% of the 8.6% per K increase in 99th percentile TC precipitation rates in global rotating radiative-convective equilibrium (RCE) simulations, with the thermodynamic changes making up majority of the rest of the contribution. These results agree with other modeling studies that suggest the thermodynamic increase in atmospheric moisture accounts for most of the extreme precipitation increases with temperature^24–26,35^, but especially in the case of TCs, it is important to keep changes in intensity in mind since the thermodynamics and convection within TCs are linked through their secondary circulations.

Although TCs can produce 40–50% of annual precipitation and over 80% of extreme precipitation in regions of the tropics^36–38^, the only study the authors could find that focused on the apparent scaling of TC precipitation was Traxl et al.^21^. Most previous literature has focused on estimating the climate scaling of TC precipitation using the difference in mean precipitation and SST between historic and future climate model simulations, and no one has attempted to understand the relative magnitudes of the apparent and climate scaling rates of TC precipitation. In some previous studies, the apparent scaling rate has been used to project how extreme precipitation may respond to warming from climate change, but these projections are invalid if the apparent and climate scalings are not approximately equal. To the authors’ knowledge, this is the first study that applies scaling rate estimation techniques typically used for general extreme precipitation to TC extreme precipitation and compares these scaling rates for TC precipitation between models and observations. We also test the sensitivity of the apparent and climate scaling rates to different precipitation metrics, since in previous studies of both TC and non-TC extreme precipitation, many different extreme precipitation metrics are used and could partially explain differences in scaling rates. The goals of this paper are to:

1. estimate the apparent scaling between TC precipitation and SST in observations and climate models to investigate if the models are capturing this relationship and to assess the dependency of the scaling rate on precipitation metric.
2. explore if idealized RCE model simulations can be used to estimate the climate scaling of TC precipitation in more realistic model simulations.
3. compare the relative magnitudes of the apparent and climate scaling rates of TC precipitation to determine if the observed apparent scaling can be used to estimate the climate scaling.

The precipitation observations are from the Integrated Multi-satellitE Retrievals for GPM (IMERG) algorithm^39^ while the SST observations are daily data from the NOAA Optimum Interpolation Sea Surface Temperature (OISST), version 2, database^40,41^. TC observed tracks and intensities are from the International Best Track Archive for Climate Stewardship (IBTrACS) database^42^. The realistic climate model simulations are two Community Atmosphere Model, version 5 (CAM5), runs. One, known as “AMIP Historical”, is run using observed climate conditions for 1980–2012 while the other, known as “AMIP Future”, is run under projected climate conditions for 2070–2099 under the RCP8.5 scenario. The idealized simulations, known as “RCEMIP”, are also CAM5 runs but are run using RCE conditions with globally-uniform SST ranging from 299–305 K. Please see the section “Datasets” for more details about all the datasets.

## Results

### TC SST environments and intensities

Before comparing TC precipitation between the models and observations, we will examine some of the general characteristics of the TCs in the datasets. First, the analysis is limited to only include TCs between 5^∘^ and 20^∘^ latitude in both hemispheres in order to focus on the region where the TC characteristics in the RCEMIP simulations, AMIP simulations, and observations are most likely to be similar. The RCEMIP simulations do not have extratropical storms or circulations, so TCs act in ways that are different from the real world poleward of about 25^∘^ latitude^13,43^. Also, this 5 to 20^∘^ latitude band is where TCs usually form and develop. Poleward of these latitudes, TCs (and their precipitation) in the AMIP simulations and observations are more likely to be influenced by strong vertical wind shear, SST gradients, and landfall. These factors impact TC precipitation patterns and distributions, which may change the scaling rates, but for this study, we wanted to focus on just the impact of SST on TC precipitation. There is some evidence that TC activity should shift towards the poles as the climate warms^44–46^, which could impact the region of TC activity between the AMIP Historical and AMIP Future simulations. However, the mean TC locations and genesis latitudes in AMIP Historical and AMIP Future, when only including storms between 5^∘^ and 20^∘^ latitude, are only different by 0.2^∘^ and 0.3^∘^, respectively.

**Fig. 1:**
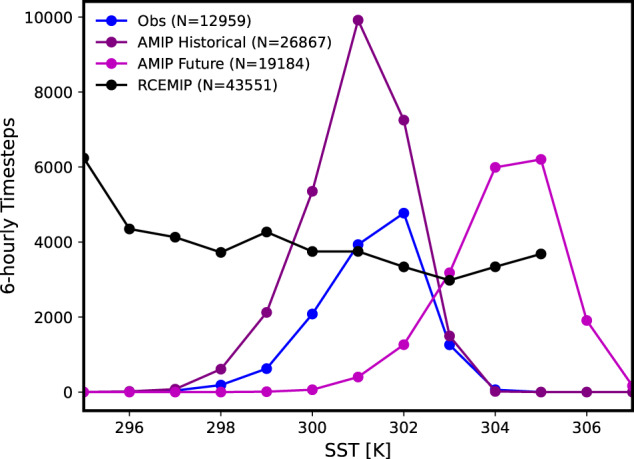
Sample sizes of TCs over different TC-local SSTs. Counts of 6-hourly timesteps when TC centers were over TC-local SSTs ranging from 295 to 307 K for observations (blue), AMIP Historical (purple), AMIP Future (pink), and RCEMIP (black). For the observations and the two AMIP simulations, the TCs are binned into 1 K bins centered on each SST value (e.g., the 300 K bin includes 299.5 K to 300.5 K). The numbers in the legend show the total summed counts for each dataset. Only TCs with center locations over the ocean and between 5^∘^ and 20^∘^ latitude in both hemispheres are included.

Figure 1 shows the counts of 6-hourly timesteps when the TCs’ centers were over different SSTs. TCs preferentially exist over SSTs between 299 and 303 K in observations and AMIP Historical. The mean SST for observations is about 0.4 K warmer than the mean SST in AMIP Historical. It is important to note that the datasets are different lengths, which explains most of the differences in counts at the same SSTs between the observations and AMIP Historical as well as the 0.4 K difference in the means. If the counts are plotted for the common time period of 2001–2012 only (not shown), the observed and AMIP Historical counts are almost identical and the means differ by less than 0.1 K. The AMIP Future mean SST is 3.3 K higher than the AMIP Historical mean, which is larger than the difference between the global mean SSTs of 3.1 K and smaller than the difference between the 5^∘^–20^∘^ latitude means of 3.6 K. The standard deviations of the TC-local SST distributions are 1.09 K for Observations, 1.09 K for AMIP Historical, and 1.17 K for AMIP Future, demonstrating that the variability of TC-local SSTs is similar between these datasets. TCs in the RCEMIP simulations occur at all simulation SSTs, resulting in the highest total number of timestep counts (noted in the legend) even with less than 2 years of simulation time. This is one advantage of using idealized modeling: more TCs can be generated than in realistic simulations at the same horizontal grid spacing while using less computational resources. This is helpful when analyzing extreme events, like TCs, that are relatively rare in the real world. This provides motivation for assessing whether or not the RCEMIP simulations have similar TC precipitation scaling rates as the observations and AMIP simulations because if they do, we may be able to use them to project future changes in TC precipitation at relatively low computational cost.

**Fig. 2:**
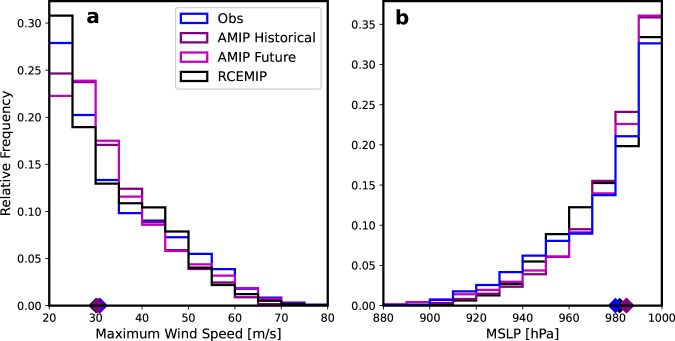
Distributions of two measures of TC intensity. Relative frequencies of TC intensities, measured by the **a** maximum low-level wind speed [m/s] and **b** minimum sea level pressure [hPa], for observations (blue), AMIP Historical (purple), AMIP Future (pink), and RCEMIP (black). Only TCs over the ocean with center locations between 5^∘^ and 20^∘^ latitude in both hemispheres are included. Medians of the distributions are marked by the diamonds on the x-axis.

TC intensities are known to influence their precipitation rates, especially in the TCs’ inner cores^47,48^. Figure 2 shows the distributions of relative frequencies of TC intensities, measured by both the maximum low-level wind speed in m/s and the minimum sea level pressure (MSLP) in hPa. To be as consistent as possible with observations, the 10-meter maximum wind speed was estimated for the RCEMIP, AMIP Historical, and AMIP Future output from the lowest model level wind speed using a logarithmic law with an open sea roughness coefficient^49^. This results in a reduction factor of about 15% from CAM5’s lowest model level, which is about 60 m. Looking at the maximum wind speed distributions (Fig. 2a), the shapes of all the distributions are similar, and the medians (diamonds on the x-axis) all cluster between 30 and 31 m/s, with the largest difference between any two medians being 2.9%. Only TCs with intensities greater than or equal to 20 m/s are included in the median calculations and in the subsequent TC precipitation analysis. While the AMIP Historical and AMIP Future medians of the maximum wind speed distributions are very similar, the median of the AMIP Future lifetime maximum wind speed distribution is about 5% (1.5% per K) higher than the AMIP Historical median (not shown). This is consistent, albeit on the low side of the range, with the future projections of TC lifetime maximum intensity from Knutson et al.^33^ of 0.5–5% per K. Looking at the MSLP distributions (Fig. 2b), the observed distribution has the lowest median of 980.0 hPa, followed by RCEMIP at 981.8 hPa, AMIP Future at 984.6 hPa, and AMIP Historical at 984.9 hPa. The distributions again have similar shapes and medians, with a maximum 0.5% difference between any two medians. While the TC intensity distributions in Fig. 2 do not match exactly between the different datasets, this is to be expected to an extent since 10-meter wind speeds in models are not direct comparisons to those from IBTrACS. Model wind speeds have different averaging periods and must be adjusted down to 10 meters from the lowest model level. It has also been shown that TC intensities do not impact TC precipitation scaling rates nearly as much as thermodynamic warming^13,50^, so any small differences in TC intensity distributions are unlikely to greatly affect the overall results.

### SST dependence of TC precipitation

**Fig. 3:**
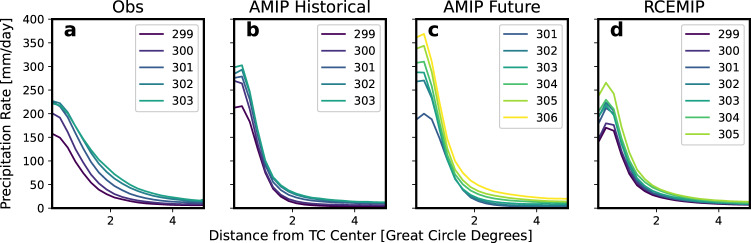
Radial profiles of TC precipitation over different TC-local SSTs. Mean radial profiles of 6-hourly TC precipitation rates [mm/day] for different SSTs for **a** observations, **b** AMIP Historical, **c** AMIP Future, and **d** RCEMIP. For the observations and the two AMIP simulations, the TCs are binned into 1 K bins centered on each SST value (e.g., the 300 K bin includes 299.5 K to 300.5 K), and different colored profiles represent the different SSTs as noted in the legends.

Focusing on precipitation characteristics, the mean radial profiles of the 6-hourly TC precipitation rates separated by TC local-SST are shown in Fig. 3. The profiles are only plotted for SSTs that have at least 100 samples, and these are the SSTs for each dataset that are used for all TC precipitation analysis. In all of the radial profiles, precipitation rates decrease with increasing distance from the TC center, as expected based on previous work on TC precipitation structure^47^. The AMIP Historical inner-core precipitation magnitudes are larger than the observed values for the same SSTs. This has been demonstrated for TCs in CAM5 in at least one previous study^29^ and could be related to the relationship between the model’s physics timestep and the partitioning between large-scale and parameterized precipitation in CAM5^51,52^. Moon et al.^53^ also found higher peak inner-core 6-hourly precipitation rates compared to observations in HighResMIP model simulations^54^ with similar grid spacings as the AMIP simulations in this study. For the common SSTs between AMIP Historical and AMIP Future (301–303 K), the peak precipitation rates are lower or about the same for AMIP Future. This may be because the background SST in AMIP Future is warmer than in AMIP Historical by about 3 K, but weak TCs still exist (Fig. 2). While TCs in AMIP Historical over 301–303 K SSTs are likely to be more intense since these are very warm SSTs for the current climate, there are less and weaker TCs over this SST range in AMIP Future. Another possible explanation is an increase in atmospheric stability in the future climate^30,55^ over this SST range, which could decrease extreme precipitation rates in the AMIP Future storms compared to AMIP Historical storms. In all datasets, increasing SST leads to increases in precipitation rate. The change in precipitation rate, however, is not uniform in space: changes in the TC inner-core appear larger than changes farther from the TC center, especially in the models. The observations suggest a more uniform increase in precipitation rate with SST. The response of inner-core precipitation to SST warming in the observations in Fig. 3 appears to contradict the decreasing trend in average inner-core precipitation rates found using about 20 years of TRMM data^27,56,57^. However, we argue these results do not disagree with each other but instead suggest that the present-day relationship between inner-core TC precipitation and SST is different than the pattern of TC precipitation structure change over time. It is also possible that our results are different due to different precipitation and SST datasets, analysis time periods, and latitude limits.

**Fig. 4:**
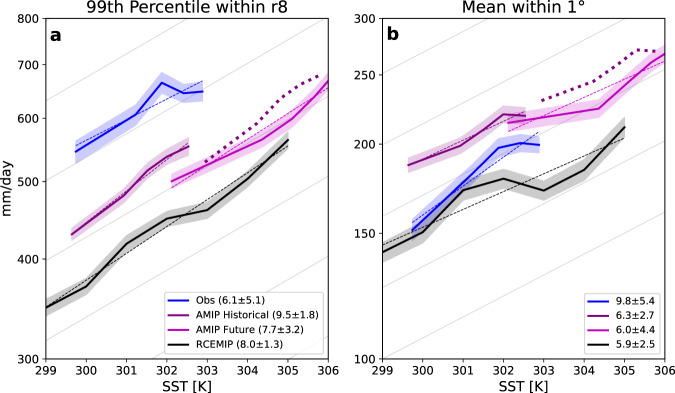
Two metrics of TC precipitation and their relationship to TC-local SST. **a** 99th percentile of 6-hourly TC precipitation rates within r8 and **b** mean of 6-hourly TC precipitation rates within 1^∘^ of the TC center, binned by SST, for observations (blue), AMIP Historical (purple), AMIP Future (pink), and RCEMIP (black). The y-axis is on a logarithmic scale, and the units of precipitation rate are mm/day. Values are binned into 5 SST bins that each have approximately the same number of samples. The thin dashed colored lines show the exponential regression fit. The light gray lines in the background show the C-C rate. The thick dotted purple line shows the AMIP Historical line multiplied by 0.07 and by the difference in global mean SST between AMIP Historical and AMIP Future (3.3 K). The shaded regions behind the lines show the 95% confidence intervals, calculated using bootstrapping with 10,000 repetitions with replacement. Numbers in the legends are the apparent scaling of the precipitation metric, shown with 95% confidence intervals estimated using a student’s t-distribution.

Figure 4 shows the relationship between two metrics of 6-hourly TC precipitation rates (99th percentile within the radius of the 8 m/s tangential wind speed, r8, in panel a and mean within 1^∘^ from the TC center in panel b) and SST. These plots are motivated by Figure 4 in Zhang et al.^58^, and details of how the scaling rates are calculated are in the section “Scaling rate calculations”. Looking at the magnitudes of the precipitation metrics, there is some spread between the datasets, with observations having the highest 99th percentiles consistently. This may seem to contradict Fig. 3, but looking at the mean precipitation within 1^∘^, the observational values are smaller than AMIP Historical. This shows that IMERG has higher extreme precipitation values than AMIP Historical but lower precipitation averaged within the TC inner-core. The RCEMIP simulations have the lowest magnitudes for both metrics across all SSTs. This may seem counter-intuitive since TCs in the idealized RCEMIP simulations have similar intensities as the AMIP simulations and observations (Fig. 2) and do not encounter SST gradients or high wind shear. More work comparing TC precipitation in RCEMIP simulations to observations is needed but is beyond the scope of this study.

The slopes of the lines in Fig. 4 represent the apparent scaling of each precipitation metric. For visual comparison, the light gray lines in the background show the C-C rate (7% per K). The estimated apparent scaling rate for each line and the 95% confidence intervals for the rate are noted in the plot legends, and an explanation of the calculation of these values is in the section “Scaling rate calculations”. For the 99th percentile TC precipitation, the observed apparent scaling is the smallest at 6.1% per K while the largest is AMIP Historical at 9.5% per K. AMIP Future and RCEMIP have similar scaling rates at 7.7% and 8.0% per K, respectively. For the mean precipitation within 1^∘^, the observed scaling rate is the largest at 9.8% per K while the smallest is RCEMIP at 5.9% per K. AMIP Historical and Future are closer to the RCEMIP rate at 6.3% and 6.0% per K, respectively. These results show that the apparent scaling of TC precipitation can depend on precipitation metric. While the apparent scaling rates do not match exactly between observations and models, their 95% confidence intervals overlap. Note that the 8.0% per K scaling rate for 99th percentile precipitation in RCEMIP is different than the 8.6% per K value from Stansfield and Reed^13^. This is because the rates are calculated in slightly different ways and because in this paper the latitude limits for the analysis are 5^∘^–20^∘^ while in the previous paper they were 40^∘^S–40^∘^N.

In the Supplementary Information, we explore how the apparent scaling can depend on the TC precipitation extraction radius, the exclusion or inclusion of non-precipitating points, and the choice of SST bins. Generally the apparent scaling is the most consistent between all the variations for the RCEMIP simulations, likely because the TCs in those simulations do not encounter many factors that impact TC precipitation in the real world, like large vertical wind shear or SST gradients. The apparent scaling rates between AMIP Historical and AMIP Future tend to be fairly similar, varying by a maximum of 4.4% per K across the methodologies. The observational scaling rates vary depending on the analysis choice but range between around the C-C scaling to just over double the C-C scaling. This variability in apparent scaling rate with different analysis choices is unsurprising in light of Fig. 3. If the change in TC precipitation with SST varies in the radial dimension, then any metric that uses a spatial averaging length would indicate different scaling rates with different averaging lengths.

The slope between the AMIP Historical and AMIP Future lines in Fig. 4 represents the climate scaling between those two simulations. Where the AMIP Future line is in relation to the AMIP Historical line shows how the magnitude of the climate scaling relates to the magnitude of the AMIP Historical apparent scaling (see Figure 3 in Zhang et al.^58^ for a schematic explanation). The thick dotted purple line shows the AMIP Historical line multiplied by 0.07 (i.e., the C-C rate) and by the difference in the mean SSTs under TCs between AMIP Historical and AMIP Future (3.3 K), which shifts the line by 7% per K. If the AMIP Future results were to exactly line up with this dotted purple line, this would indicate that the climate scaling of the AMIP simulations is following C-C. The AMIP Future line is shifted right downward compared to the AMIP Historical line for both precipitation metrics, indicating the apparent scaling is larger than the climate scaling, and is slightly below the dashed purple line, meaning the climate scaling should be below C-C. Note that since the apparent scaling is larger than the climate scaling and the AMIP Future line is shifted right downward compared to the AMIP Historical line, it is expected that the AMIP Future line will be below the AMIP Historical line at their common SSTs. The climate scaling is 4.9% per K for 99th percentile precipitation, with a range from 4.2 to 6.4% per K, and 4.7% per K for mean precipitation within 1^∘^, with a range from 3.6 to 6.1% per K (see section “Scaling rate calculations” for details of the calculation). If the climate scaling is instead calculated using the percentage difference between the medians of all precipitation within r8 for AMIP Historical and AMIP Future, which is the method used in many previous studies of TC precipitation scaling^33^, the rate is 5.0% per K. One reason the climate scaling may be less than the C-C scaling is because in AMIP Future, the tropical-mean atmospheric moisture deficit is larger than in AMIP Historical (not shown), meaning this relatively drier air may be ventilated into the TCs in AMIP Future and decrease their precipitation rates.

If the climate scaling is calculated for RCEMIP by splitting the dataset into cooler and warmer halves (i.e., 299–302 K and 302–305 K), the rates are 7.7% per K for 99th percentile and 5.1% per K for mean precipitation, which are within the confidence intervals for the apparent scaling for both metrics. These values are both larger than the climate scaling in the AMIP simulations, with the value for mean precipitation within 1^∘^ being more similar. It is possible that the larger climate scaling in RCEMIP is caused by different TC characteristics compared to the AMIP simulations, such as the greater increase in lifetime maximum intensity with increasing SST or the larger mean size of the outer TC circulation (not shown). The apparent scaling rate for the 299–302 K RCEMIP simulations is 9.3% per K for 99th percentile precipitation and 8.8% per K for mean precipitation. For 302–305 K, the 99th percentile apparent scaling is 8.0% per K and the mean precipitation apparent scaling is 5.8% per K. For both TC precipitation metrics, the apparent scaling is lower for the 302–305 K simulations compared to the 299–302 K simulations. This is consistent with the change in apparent scaling when comparing the AMIP Historical to AMIP Future; therefore, both the RCEMIP and AMIP simulations suggest that the apparent scaling of TC precipitation may decrease in the future as SSTs warm.

## Discussion

As air and ocean temperatures warm due to climate change, it is expected that extreme precipitation will increase over most of the globe^59^. Understanding how changes in the storm systems that produce extreme precipitation will impact the precipitation itself is challenging. A lack of long-term, high-resolution observations over the tropical oceans has made this task especially difficult for TCs. This work explores two TC precipitation scaling rates in the context of CAM5 simulations and observations: (a) the apparent scaling, which is the present-day variability between TC precipitation and SST and (b) the climate scaling, which is the long-term response of TC precipitation to SST warming. The main results of this paper are:

- The apparent scalings of TC precipitation are all around the Clausius–Clapeyron rate of 7% per K and compare well between models and observations.
- The climate scaling is estimated to be about 5% per K, smaller than the apparent scalings and the Clausius–Clapeyron rate.
- Scaling rate values can depend on various analysis choices, such as precipitation metric and averaging radius around the TC center.

All of the apparent scaling rates for TC precipitation calculated in this work are positive. This is different than the negative rates found in Traxl et al.^21^ when only TC precipitation was isolated and is likely a result of the temporal frequency of the SST datasets. Their argument is that the broad TC wind fields and large-scale cirrus cloud cover lead to SST cooling that precedes the storm itself and its heaviest precipitation rates, resulting in a negative apparent scaling between TC precipitation and SST. While Traxl et al.^21^ used hourly SSTs from ERA5, this analysis used daily observed SSTs. Cold wakes from previous TC passages do appear in daily mean SSTs^60^, but daily mean SSTs are not likely to see as large of a preceding cooling effect from outer TC winds and cloud cover as hourly SSTs, which could explain the discrepancy between the results presented here and in Traxl et al.^21^.

The apparent scaling rates depend on precipitation metric and other analysis choices (see Supplementary Table 1). The authors want to emphasize that these results are not implying that one TC precipitation metric is better than any others, but that future work should choose precipitation metrics based on their specific application^61^ and consider using multiple metrics to see if their results are consistent for various metrics. The apparent scaling rate ranges between 6.1 and 9.5% per K for the 99th percentile within r8 and 5.9 and 9.8% per K for the mean within 1^∘^ from the TC center. For the observations, the rate is smaller for 99th percentile than for mean storm inner-core precipitation (within 1^∘^), but the opposite is true for all of the model simulations. Xi et al.^62^ calculated the scaling of TC precipitation within 600 km from the storm center in Weather Research and Forecast (WRF) model simulations with 3 km grid spacing. These simulations had domain-constant and time-invariant SSTs ranging from about 299 to 307 K. They found a TC precipitation scaling of 9% per K, which is similar to the RCEMIP apparent scaling of 8% per K for 99th percentile precipitation although their study used a different precipitation metric and different model set-up.

The climate scaling calculated between the AMIP Historical and AMIP Future was just under 5% per K for both 99th percentile and mean TC precipitation within 1^∘^. While the CAM5 AMIP TC precipitation climate scaling rate is less than the apparent scaling in the model simulations and observations, there is no clear theoretical model or framework linking the apparent and climate scaling rates, so it is difficult to argue whether we expect this qualitative relationship to hold in observations and other models. Previous work using a hindcast attribution approach for Hurricanes Florence and Dorian found that climate change increased the total precipitation accumulations from these storms by about 5–7% per K^63,64^; however, accumulated precipitation may be influenced by other TC characteristics that do not influence inner-core extreme precipitation rates as much, like storm translation speed, so the climate scaling for accumulated precipitation may be different than extreme precipitation rates. When using a similar methodology on the entire 2020 North Atlantic Hurricane season, Reed et al.^65^ found increases in 3-hourly 99th percentile precipitation rates of about 14% per K for all storms of at least tropical storm strength. Liu et al.^32^ used current and future climate simulations with the HiFLOR model and estimated increases in mean 6-hourly TC precipitation rates within 100 km of the center (comparable to the mean 6-hourly precipitation rates within 1^∘^ used in this paper) of 13 to 17% per K, dependent on ocean basin. This climate scaling is more than double the one estimated with the CAM5 AMIP simulations presented here, but this may be because the HiFLOR simulates 2.0–5.4% per K increase in mean TC intensity while our change in mean intensity between AMIP Historical and Future is about 1% per K. Their HiFLOR simulations were also coupled to an ocean model while the CAM5 AMIP simulations were forced with SST boundary conditions. Our results suggest that the climate scaling of TC precipitation has a smaller magnitude than the apparent scaling of TC precipitation, and this result is the same for both precipitation metrics analyzed in this paper. A potential explanation for the smaller climate scaling is an increase in the atmospheric moisture deficit throughout the troposphere in warmer climates (not shown). This indicates that using the apparent scaling to project future changes in TC precipitation in response to long-term climate warming would be inaccurate, since the apparent and climate scaling rates are different.

When studying the response of TC precipitation to warming, it is important to clarify which TC scaling rate (apparent or climate) is being estimated. In the case of RCEMIP simulations with fixed SSTs, it is not clear which scaling rate is being studied by comparing precipitation rates between runs with different SSTs. The TCs do not experience SST variability so the scaling rate is not the apparent scaling rate. On the other hand, the TCs only respond to total-domain SST warming and do not experience other changes that are expected to occur with climate change and impact TC precipitation, such as relative SST change in the tropics^66,67^ and shifts in wind shear^68^. This means that the scaling rate calculated from these RCEMIP simulations is not exactly a climate scaling either. When estimating the climate scaling by breaking the RCEMIP simulations into warmer and cooler halves, the scaling rates are larger than the climate scaling estimated using the AMIP simulations, suggesting that the RCEMIP simulations may overestimate TC precipitation’s response to warming SSTs. While the authors are not arguing that RCEMIP simulations are not useful to study TCs and their precipitation, it is important to consider what these types of simulations can imply about the real world.

As the high-resolution observational record of TC precipitation lengthens, more insights will be gained about its sensitivity to climate change. To try to understand this sensitivity at present, model simulations are beneficial, but do have limitations. Global climate models with resolutions of tens of kilometers have parameterized convection, which may cause unrealistic responses of precipitation to climate warming. It also may be important for models to have coupled ocean models in order to accurately simulate the relationship between TC precipitation and SST. TCs typically have a cooling effect on SSTs due to their high winds mixing up cooler ocean water from below the surface^60^, and this cooling impacts precipitation rates within the storm^69^. This effect of TCs on SSTs is likely not fully captured in the SST boundary conditions used to force AMIP-style (or RCEMIP-style) runs. Future work should include exploring the relationship between SST and TC precipitation in fully-coupled model runs and in next-generation global models without parameterized convection.

In summary, CAM5 shows limitations in the ability to represent the TC precipitation apparent scaling with SST when compared to observations, but all of the rates are in the same magnitude range (within 4% per K) and their 95% confidence intervals overlap. More work is needed to examine what is causing these biases. It is possible that these results could be specific to CAM5; therefore, future work could entail extending this work to different climate models. For all of the datasets, the apparent scaling rates varied based on various analysis choices, such as averaging radius; therefore for future work on TC precipitation scaling, the authors recommend using multiple precipitation metrics or focusing on specific metrics that would be most useful for hydrologic models that can project TC flooding. The climate scaling calculated from the two CAM5 AMIP simulations was just under 5% per K for both precipitation metrics used in this paper. Based on CMIP6 model projections of the global mean SST in 2071–2100 under the SSP245 and SSP585 scenarios, the multi-model mean SST will be about 1.5 K or 2.8 K warmer, respectively, than the global mean SST in 1985–2014 in the CMIP6 Historical simulations^70^. Using our estimate of the TC climate scaling, we expect extreme precipitation rates and mean inner-core rates in TCs to increase by 7.5% (i.e., 5% per K * 1.5 K) for SSP245 or 14.0% (i.e., 5% per K * 2.8 K) for SSP585 by the end of the century. That increase would be the change from the background increase in temperatures due to climate change, but it is important to mention that some TCs may see larger increases in precipitation rates due to SST variability (i.e., the apparent scaling) or changes in other characteristics that influence TC precipitation, such as TC translation speed or vertical wind shear. How much precipitation from TCs increases in the future has important implications for coastal communities and infrastructure around the world.

## Methods

### Datasets

Six-hourly TC track and intensity observations are sourced from the International Best Track Archive for Climate Stewardship (IBTrACS) database^42^. The SSTs are from the NOAA Optimum Interpolation (OISST), version 2, database^40,41^, which has daily temporal resolution and 0.25^∘^ spatial resolution. It incorporates SST data from ships, buoys, and a satellite radiometer. The precipitation data is from the Integrated Multi-satellitE Retrievals for GPM (IMERG) algorithm^39^. The data is derived from multiple satellite passive microwave sensors from the GPM constellation, and the final run is calibrated using monthly rain gauge accumulations. The product is available on a 0.1^∘^ spatial grid at half-hourly time intervals. For this analysis, the half-hourly data is converted to 6-hourly to match the other datasets. Observational analysis is limited to 2001–2020 due to the availability of IMERG.

The realistic model simulations are completed with the Community Atmosphere Model (CAM), version 5^71^. The first is an Atmospheric Model Intercomparison Project (AMIP)^72^ simulation forced with observed SSTs^73^ from 1980 to 2012. It will be known as “AMIP Historical" throughout this paper. This simulation has been used previously to study the impact of the model dynamical core on TCs^74^ and the impact of dust on North Atlantic TCs^75^. The second is a simulation run under the RCP8.5 for 2079–2099, which has been utilized, in addition to the AMIP Historical simulation, to understand how TCs and their precipitation may change in the future^76^. This simulation was forced with bias-corrected SSTs from a fully-coupled Community Earth System Model (CAM’s parent model) simulation run under the RCP8.5 scenario. More details about the SSTs used to force this future CAM5 simulation and the characteristics of TCs in the simulation can be found in Bacmeister et al.^76^. For convenience, this RCP8.5 simulation will be referred to as “AMIP Future". The horizontal grid spacing for both AMIP Historical and AMIP future is about 28 km globally. All output is converted to 6-hourly temporal frequency.

The idealized model simulations are described in detail in Stansfield and Reed^13^. They are also CAM5 simulations but run in a state of radiative-convective equilibrium (RCE). These simulations followed the protocols of the Radiative Convective Equilibrium Model Intercomparison Project (RCEMIP)^77^ and utilized the preset Community Earth System Model (CESM) compset^78^. The only modification made to the CESM RCEMIP compset is that rotation is added by setting the planetary rotation rate to that of the real Earth. The simulations have no diurnal or seasonal cycles, and the SST is globally-uniform. SSTs vary between 295 and 305 K in 1 K increments, adding up to a total of 11 simulations, although only the 299 to 305 K simulations are used for this analysis to match the SSTs found under the TCs in observations and the realistic model simulations. The horizontal grid spacing is approximately 28 km over the whole globe, the same as the two AMIP simulations. The simulations are run for 2 years with the first 2 months discarded to allow for spin-up time. Similar simulations have been used to study planetary dynamic controls on TC structure^43,79,80^ and the impact of SST on TC counts and intensities^55,81^. The model output and TC track data are publicly available^82^. The data are converted from 3-hourly to 6-hourly output. This dataset will be referred to as “RCEMIP".

### TC tracking and precipitation extraction

All TC tracking, precipitation extraction, and compositing are performed using the TempestExtremes software package^83,84^. TC tracking parameters are detailed in Stansfield and Reed^13^ for the RCEMIP simulations. For AMIP Historical and Future, tracking settings are based on those used in Bacmeister et al.^76^. Bacmeister et al.^76^ used a different TC tracker, so an effort was made to adjust TempestExtremes parameters to ensure that annual TC counts closely match those in Bacmeister et al.^76^. The annual mean TC count for AMIP Historical using our TempestExtremes settings is 70 compared to 71 in Bacmeister et al.^76^. For AMIP Future, our settings produce an annual mean TC count of 56 compared to 58 in Bacmeister et al.^76^. The command line used to track TCs in the AMIP Historical and Future simulations using TempestExtremes is reproduced below:


./DetectNodes --in_data_list "*$*infiles" --timestride 2



--in_connect *$*connectivityfile --out “*$*outfiles"



--closedcontourcmd “PSL,400.0,3.0,0;_DIFF(Z200,Z500),-10.0,2.0,1.0"



--mergedist 5.0 --searchbymin PSL



--outputcmd “PSL,min,0;_VECMAG(U10,V10),max,2;PHIS,max,0"



./StitchNodes --format “lon,lat,slp,wind,phis" --range 4.0



--mintime “72h" --maxgap 0 --in “*$*outfiles" --out “*$*trackfilename"



--threshold “wind,>=,17.0,11;lat,<=,50.0,10;



lat,>=,-50.0,10;phis,<=,150.0,10"


TC precipitation extraction is performed using the radius of the 8 m/s tangential wind speed (r8) as in Stansfield et al.^85^. For the observations, r8s are calculated using 10-meter wind output from ERA5^20^, again as in Stansfield et al.^85^. Precipitation extraction and subsequent scaling analysis is also done using a 5^∘^ great-circle distance (GCD) and a 1^∘^ GCD to compare the impact of using different extraction radii. At each timestep in each TC’s lifetime, extracted TC precipitation is re-gridded onto a common 0.25^∘^ grid centered on the TC center location using TempestExtreme’s NodeFileComposite function. This 0.25^∘^ grid is chosen to match the 28 km spacing of the CAM5 native grid. For observations, AMIP Historical, and AMIP Future, only timesteps when the TCs’ center points are over the ocean are included. This choice is made to isolate the TCs that are likely to be most similar to TCs in the RCEMIP simulations, since landfall can greatly impact the structure and precipitation of a TC^47,86^.

### TC local-SST calculation

To calculate the SST local to the TC, TempestExtreme’s NodeFileComposite function is used again. All SST data is re-gridded onto the same 0.25^∘^ common grid, centered on each TC’s center location output from the TC tracker or from IBTrACS observations and that extends out about 5^∘^ in each direction. The SST is then averaged over this spatial grid at each timestep to calculate the TC local-SST. The sensitivity of results to the size of the SST composite grid was tested by halving the size of the grid, and the results were impacted very little.

### Scaling rate calculations

Before calculating the scaling rates, the TC precipitation is first binned by the TC local-SST. The data are divided into 5 bins of approximately equal sample size, and a precipitation metric (i.e., either 99th percentile or mean) is calculated for each bin. Note that when calculating the precipitation metrics, only precipitating points (values greater than 0 mm/day) are included. The apparent scaling rate of TC precipitation is estimated using an exponential regression by fitting a least-squares linear regression to the logarithm of the precipitation metrics across the 5 bins. The middle of each SST bin is used in the regression. This method is commonly used to estimate the apparent extreme precipitation-temperature scaling^6,18,21^. To estimate the climate scaling, a methodology very similar to that described in Zhang et al.^58^ is utilized. Where *P*_*Hist*_ is the precipitation metric in a specific bin from AMIP Historical, *P*_*Fut*_ is the AMIP Future precipitation metric in the same bin, *δT* is the difference in SST between AMIP Historical and AMIP Future in that bin, and *β* is the climate scaling rate for that bin, $\log \frac{{P}_{Fut}}{{P}_{Hist}}={(1+\beta )}^{\delta T}$. *β* is calculated for each of the 5 bins, and the median is reported as the climate scaling between AMIP Historical and AMIP Future.

### Supplementary information

Supplementary Material

## Data Availability

The International Best Track Archive for Climate Stewardship (IBTrACS)^42^ database is available online at https://www.ncei.noaa.gov/products/international-best-track-archive. The NOAA Optimum Interpolation Sea Surface Temperature (OISST), version 2, database^40,41^ is available at https://www.ncei.noaa.gov/products/optimum-interpolation-sst. Integrated Multi-satellitE Retrievals for GPM (IMERG) data^39^ is available for download at https://gpm.nasa.gov/data/imerg. ERA5^20^ data is available for download at https://www.ecmwf.int/en/forecasts/datasets/reanalysis-datasets/era5. The CAM5 AMIP simulation (Historical and Future) output is stored on the National Energy Research Scientific Computing Center’s High-Performance Storage System (HPSS), available via Globus Endpoint. The CAM5 RCEMIP simulation output is documented and available for download^82^. The CMIP6 model output used in this manuscript is available for download at https://cds.climate.copernicus.eu/cdsapp#!/dataset/projections-cmip6?tab=form.

## Code availability

All code is available upon request to Alyssa Stansfield.
