# Supplementary material for: Global tropical cyclone precipitation scaling with sea surface temperature
Source: NPJ Clim Atmos Sci. 2023 Jun 5;6(1):60. doi: 10.1038/s41612-023-00391-6 (PMC11041753; doi:10.1038/s41612-023-00391-6)
Supplement: Supplementary file 1 — Supplementary Material [file 41612_2023_391_MOESM1_ESM.pdf]

Supplemental Information for "Global Tropical Cyclone Precipitation Scaling with Sea Surface Temperature"

Alyssa M. Stansfield[1,2*] and Kevin A. Reed[1]

[1*]School of Marine and Atmospheric Sciences, Stony Brook University, 100 Nicolls Rd, Stony Brook, 11794, NY, USA.
[2]Department of Atmospheric Science, Colorado State University, 3915 Laporte Ave., Fort Collins, 80521, CO, USA.

*Corresponding author(s). E-mail(s): alyssa.stansfield@colostate.edu;
Contributing authors: kevin.reed@stonybrook.edu;

1 Supplementary Note 1: Apparent Scaling Sensitivity to Analysis Choices

Here we test the sensitivity of the TC precipitation apparent scaling rates to changes in the TC precipitation extraction radius, the exclusion or inclusion of non-precipitating points, and the choice of SST bins (Table 1). Changing the TC precipitation extraction radius from r8 to 5° for calculating the 99th percentile reduces the scaling rate for the models but increases it for observations. These changes could be due to extra non-TC precipitation being included when using a fixed extraction radius. The median r8s for ERA5 and all of the model simulations are around 400 km (not shown), so a 5° extraction radius is larger than r8 for about half of the TC timesteps. For the mean precipitation using the r8 radius instead of 1°, the scaling rates are greatly increased for observations and the AMIP simulations and increased but to a lesser degree for RCEMIP. When taking the mean precipitation within r8, precipitation in the outer part of the TCs is included. It is possible that this outer precipitation increases with SST at a faster rate than the inner-core precipitation, but this is

beyond the scope of our study. When including non-precipitation points (values equal to 0 mm/day) for 99th percentile, the scaling rate for observations more than doubles but decreases for the models. For observations, the ratio of non-precipitating points to total points going into the calculation decreases with increasing SST (not shown). This would contribute an increase to the 99th percentile with warmer SST and thus should explain some of the large increase in scaling rate. The opposite is true for the AMIP models. For the mean when including non-precipitating points, the rates do not change much, which is to be expected since there likely are not many non-precipitating points within 1° of the TC center when the data is at 0.25° grid spacing. Changing the SST binning from 5 bins with equal sample sizes to equi-width 0.5° bins increases the scaling rates to about C-C or above for both 99th percentile and mean precipitation. The RCEMIP rate does not change since each simulation has a globally-uniform SST and therefore the precipitation values are always just binned by simulation.

Supplementary Table 1: TC precipitation apparent scaling rates using different precipitation extraction radii, inclusion of non-precipitating points, and SST binning.

	Obs	AMIP Historical	AMIP Future	RCEMIP
Change in TC Extraction Radius				
99th Percentile, 5° radius	8.7	6.1	6.0	7.6
Mean, r8 radius	15.2	20.1	15.7	8.4
Change in Included Points				
99th Percentile, including 0 mm/day	15.8	5.4	5.8	7.7
Mean, including 0 mm/day	9.6	6.7	6.2	6.1
Change in SST Bins				
99th Percentile, 0.5° Bins	9.1	11.0	12.2	n/a
Mean, 0.5° Bins	14.4	7.0	9.0	n/a
